# From the Freezer to Implant: Does Cadaver Freezing Affect the Accuracy of 3D-Printed Scaphoid Prosthesis Models?

**DOI:** 10.3390/bioengineering12111183

**Published:** 2025-10-30

**Authors:** Philipp Honigmann, Mathias Haefeli, Geert Streekstra, Johannes Dobbe

**Affiliations:** 1 Hand Center Northwestern Switzerland, 4133 Pratteln, Switzerland; 2 Medical Additive Manufacturing Research Group (MAM), Department of Biomedical Engineering, University of Basel, 4123 Allschwil, Switzerland; 3 Department of Biomedical Engineering and Physics, Amsterdam UMC, University of Amsterdam, Meibergdreef 9, 1105 Amsterdam, The Netherlandsg.j.streekstra@amsterdamumc.nl (G.S.);; 4 Hand Surgery, Kantonsspital Graubünden, 7000 Chur, Switzerland; 5 Department of Radiology and Nuclear Medicine, Amsterdam UMC, University of Amsterdam, Meibergdreef 9, 1105 Amsterdam, The Netherlands; 6 Amsterdam Movement Sciences, Musculoskeletal Health—Restoration and Development, 1081 Amsterdam, The Netherlands

**Keywords:** 3D printing, additive manufacturing, cadaver, patient-specific, scaphoid bone, segmentation, three-dimensional, wrist

## Abstract

Background: Cadaveric specimens used in research are commonly frozen when stored and may undergo multiple freeze–thaw cycles during experimental procedures. To streamline workflows and minimize potential tissue degradation from repeated thawing, this study evaluated whether a frozen cadaver wrist can be reliably used to model a scaphoid for the development of a patient-specific prosthesis design method in a preclinical setting. Additionally, we assessed the impact of segmentation smoothing techniques on the accuracy of the prosthesis model. Methods: High-resolution computed tomography (CT) scans were performed on a cadaver wrist in both frozen and thawed states. Scaphoid bones were segmented using two approaches: a tight (native surface) segmentation and a smoothened version optimized for prosthetic articulation. The resulting 3D models were registered, and volume and shape differences between frozen and thawed states, as well as between segmentation methods, were quantified. Results: No statistically significant volume differences were observed between scaphoid models segmented from frozen and thawed conditions (*p* = 0.46). The average difference was 0.14% (SD 0.55). Furthermore, smoothing segmentation had minimal impact on the overall dimensions of the scaphoid model. Conclusions: Frozen cadaver wrists can be used to accurately model scaphoid bones for patient-specific prosthesis design without introducing significant volumetric deviations. Segmentation smoothing, which is necessary for prosthesis fabrication, does not compromise anatomical accuracy, supporting the feasibility of using frozen specimens for preclinical modeling.

## 1. Introduction

Patient-specific scaphoid prosthesis implantation is a valuable treatment option for non-reconstructable scaphoid bones, particularly for complex non-union fractures and advanced stages of avascular necrosis [1,2]. Early in vitro investigations into scaphoid prosthesis design have analyzed carpal kinematics to compare prosthetic motion patterns with those of healthy wrists [2], revealing that achieving accurate prosthesis sizing is critical: oversizing may lead to overstuffing of adjacent carpal joints, resulting in stiffness and a restricted range of motion, while undersizing can cause insufficient bone stability, misalignment, and carpal height loss, potentially leading to dorsal intercalated segment instability (DISI) and carpal collapse.

In vitro studies on carpal biomechanics frequently utilize cadaveric specimens. To prevent biological degradation, these specimens are typically preserved through freezing at temperatures around −20 °C, and imaging of the carpal bones for prosthetic design is usually performed in a frozen state to minimize potential tissue degradation due to repeated thawing. However, freezing can induce volumetric alterations in biological tissues due to water expulsion (where water is forced out of the tissue structure during the freezing process) and ice crystal formation [3,4,5,6,7], but previous research has demonstrated that freezing and thawing do not significantly impact the structural integrity or dimensions of larger human bones such as the skull, spine, femur, and cancellous bone [3,4,5,6,7]. Nevertheless, the human carpal bones, including the scaphoid, present a unique anatomical challenge due to their dense cortical bone layer and limited intramedullary space. This structure is also expected to have minimal capacity to compensate for volume changes caused by the thermal expansion or contraction of intramedullary fluids.

Given these anatomical differences, it remains unclear whether freezing and thawing cycles could alter the dimensions of the scaphoid bone in a clinically significant manner. If freezing does affect scaphoid morphology, prostheses modeled from CT scans of frozen specimens may not fit thawed wrists accurately, potentially compromising the validity of in vitro testing and subsequent in vivo applications. Addressing this uncertainty is essential for ensuring that prosthesis designs based on cadaveric data are anatomically precise and functionally reliable.

Therefore, the primary objective of this study was to investigate whether freezing influences scaphoid size by comparing volumetric data from CT-based models of frozen and thawed cadaver wrists. Additionally, since prosthetic surfaces require smooth articulation with adjacent cartilage, segmentation post-processing is necessary to eliminate surface irregularities. Thus, a secondary objective was to evaluate the dimensional impact of smoothing procedures on the scaphoid model. Furthermore, we employed static distance mapping to visualize and quantify surface deviations using color-coded distance maps to assess spatial discrepancies between tight (native surface) and smoothened scaphoid segmentations [8,9].

## 2. Materials and Methods

### 2.1. Specimens and Image Acquisition

**Figure 1:**
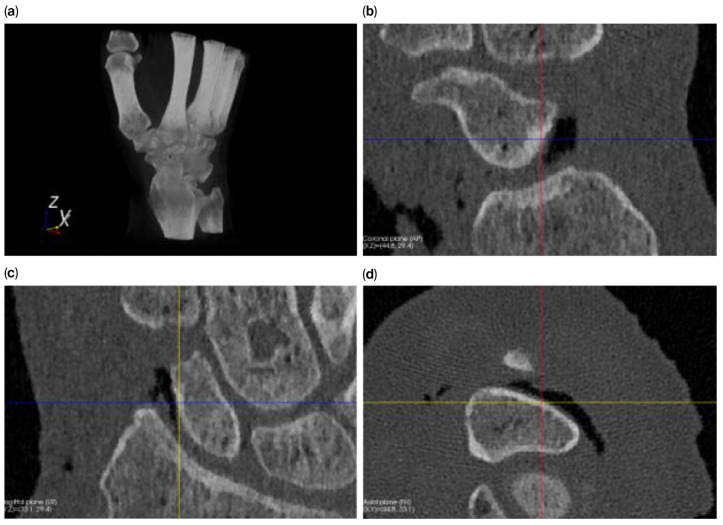
Volume rendering (**a**) and multiplanar reconstruction of the scaphoid ((**b**) = sagittal; (**c**) = coronal; (**d**) = axial).

A native healthy forearm cadaver specimen—handled in accordance with the Human Research Act (Art. 36 and 37.1 HRA)—was donated with informed consent from the donor during their lifetime. This forearm was fresh frozen at −20 °C for >3 months, and ten CT-scans in the frozen and thawed conditions (completely thawed at 21 °C room temperature) were obtained using a standard wrist protocol (120 kVp; 120 mAs; slice thickness: 0.4 mm; voxel size: 0.17 × 0.17 × 0.2 mm) on a 2 × 64 row detector dual-source computed tomography scanner (Biograph 128^®^, Siemens Healthineers, Forchheim, Germany). The arms were scanned ten times in each condition to determine variability in the volume and shape measurements of the scaphoid due to the variability in the image acquisition and segmentation. Figure 1 shows a volume rendering and multiplanar reconstruction of a CT scan.

### 2.2. Size Determination

The size of a scaphoid, in terms of its length and width, has high interindividual and gender-related differences [10]. Pichler described volumes for the determination of the size of a scaphoid [11], while Giessen described the 5 most important modes of variation in the scaphoid based on its orientation, resulting in variations between 5 and 29% depending on the view [12]. Even though there are 4 representative standard views of the scaphoid (radial, ulnar, dorsal, and volar) and more reliable techniques for size determination, such as the length-to-height ratio, it remains challenging to precisely measure the size of the scaphoid [13,14]. Therefore, for our purposes, we compared the volumes.

### 2.3. Segmentation and Volume Quantification

Regions representing the scaphoid are identified in the CT images based on CT values (Hounsfield units). For the segmentation of the scaphoid from the CT images, we used software developed in our research group [14]. A recent literature review by van Eijnatten stated that thresholding remains the most widely used segmentation method in medical additive manufacturing [15]. Therefore, threshold-connected region growing was used to start from a seed point identified by the user in the cortical layer, and a filling algorithm subsequently filled the remaining holes and closed the outline. Then, a Laplacian level-set growth algorithm was used to increase pixel dispersion to the outline of the bone, and a polygon mesh was finally extracted at the zero level of the level-set image. The volume of a segmented polygon mesh was quantified using the method described by Abdalmajeid MA and coworkers, as implemented in the Visualization Toolkit (VTK 7.1.0, Kitware Inc., New York, NY, USA), which was used in our custom software [16,17].

### 2.4. Effect of Gaussian Smoothing on Segmentation

Raw CT images may contain noise and other artifacts that can affect the segmentation process and result in errors in the 3D model of an object. Because the surface of the prosthesis articulates directly with the cartilage layer of the corresponding bone, tight segmentation is not desirable for joint surfaces because it leads to a rough and irregular surface. Therefore, we used the smoothing function to obtain a closed and smooth surface without any extra-anatomical irregularities.

**Figure 2:**
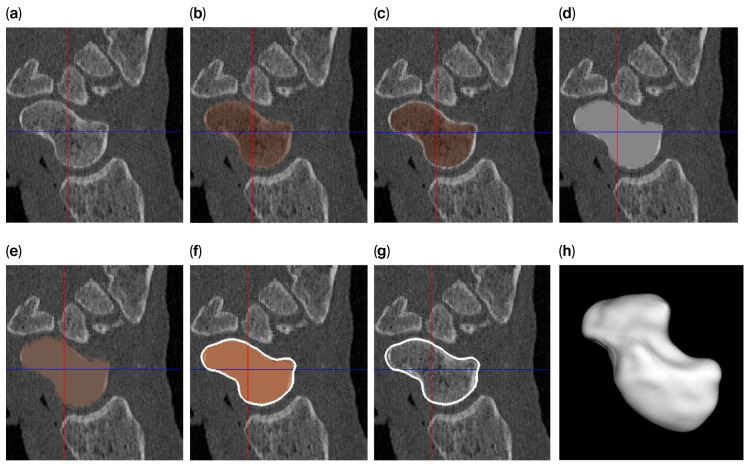
Segmentation process for a smooth 3D model of the scaphoid: (**a**) original image; (**b**) segmentation using region growing and filling; (**c**) after 0.5 mm of erosion; (**d**) after filling the segmented part of the gray-level image with a value of 2106 (the approx. value of the cortical layer); (**e**) after region growing and filling of the modified image; (**f**) polygon outline; (**g**) slight mismatch between the outline and the underlying gray-level image; and (**h**) a smooth 3D representation of the scaphoid polygon.

The level-set algorithm initially uses Gaussian smoothing to remove these irregularities, but applying such a smoothing filter to a thin cortical boundary may cause this layer to widen (caused by the point-spread function of the Gaussian filter kernel), which in turn causes the scaphoid object to become slightly enlarged after segmentation. Therefore, different segmentation approaches are used to answer the research questions (Figure 2) as follows:

- For investigating the size difference and reproducibility of segmentation between frozen and thawed conditions, image smoothing was carried out with rather limited parameters (σ = 0.3 mm; level-set iterations = 20), resulting in a tight-fitting segmentation.
- To achieve a smooth scaphoid object for implantation, further image smoothing was used (σ = 1.0 mm; level-set iterations = 30), resulting in a segmentation with a smooth outline. However, for the reasons outlined above, the object will become slightly enlarged. To circumvent this issue, we first changed the underlying gray-level image so that the cortical layer is no longer thin. This was achieved by first segmenting the scaphoid by region-growing and region-filling conditions (no level set) and subsequently eroding the segmented object by 0.5 mm. The masked (trabecular bone) region was subsequently replaced by a CT value of 2106 HU (an approximate intensity value of the cortical layer). As a result, the thin layer disappears, minimizing the widening effect. This adapted gray-level image was subsequently segmented as normal, as described above (σ = 1.0 mm; level-set iterations = 30).

## 3. Results

### 3.1. Volumetric Differences Between Frozen and Thawed Scaphoids

Tight segmenting each scaphoid 10 times for the frozen and thawed arm (i.e., using σ = 0.3 mm and 20 level-set iterations) resulted in a scaphoid polygon with carrying volumes as shown in Table 1. A paired *t*-test was conducted to compare the two measurement sets. The mean difference was 1.78 units (*SD* = 5.64), with a 95% confidence interval of [−3.43, 6.98]. This difference was not statistically significant (*t*(9) = 0.77, *p* = 0.46). The effect size was small (Cohen’s *d* = 0.24). In relative terms, the average change corresponded to only 0.14%, which is below the 0.2% threshold.

The segmentation demonstrated high reproducibility with minimal volume variation.

**Figure 3:**
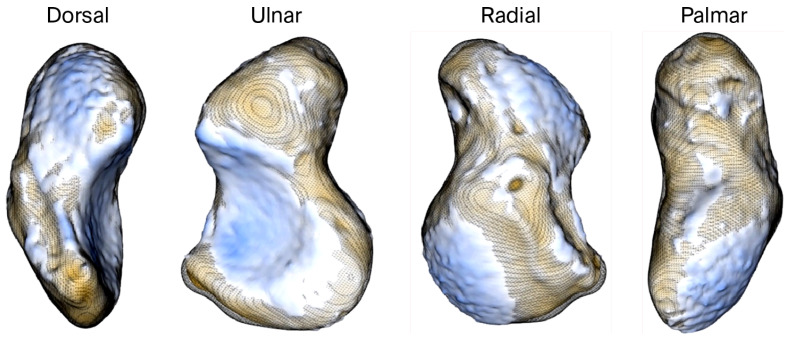
Four standard views of a signed-distance heat map representing the distance between tight/rough and smooth scaphoid segmentations. When the rough scaphoid is larger (blue surfaces), the values are positive; otherwise, they are negative (orange surfaces).

We used a signed-distance heat map to visualize (Figure 3) and quantify (Table 2) the absolute and signed distances between tight and smoothened scaphoid segmentations.

### 3.2. Differences Between Native and Smoothed Scaphoids

**Figure 4:**
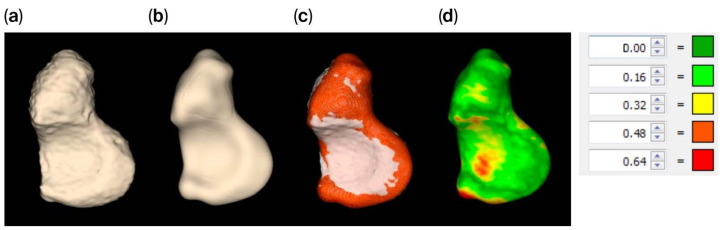
(**a**) Tight segmented scaphoid and (**b**) smooth scaphoid. The difference between the tight segmented and smoothed scaphoids, visualized by (**c**) overlaying the objects (red = smoothed), with (**d**) a heat map showing the distance between the tight and smoothed scaphoid models (values in mm).

**Figure 5:**
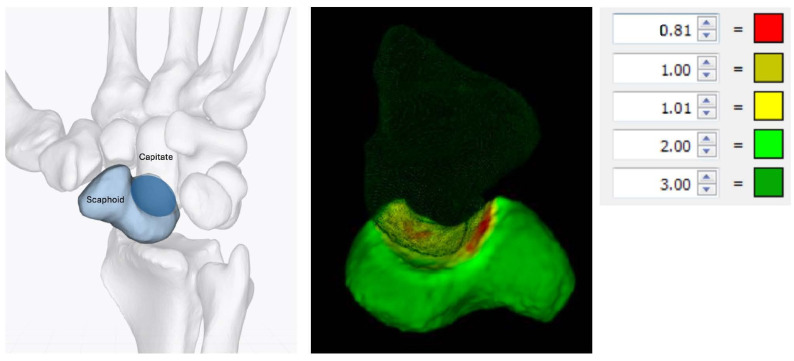
(**Left**) The dark blue area indicates the articular surface of the scaphoid with the corresponding capitate. (**Right**) A heat map showing the distance between the tight scaphoid segmentation and the capitate (i.e., cartilage space ≥ 0.81 mm). The dark red areas indicate the narrowest distances, while the dark green areas represent the largest distances.

The tight segmented scaphoid shows a slightly rough appearance (Figure 4a), while the smooth alternative (Figure 4b,c) exhibits minor local surface variations up to 0.64 mm, as indicated by the registration of the models. This variation is quantified by the heat map in Figure 4d, but the value is relatively small when compared to the distance between the scaphoid and capitate, as illustrated in Figure 5, which exceeds 0.81 mm.

The joint space thicknesses (JSTs) between the modeled scaphoid and the adjacent bones are of major interest in estimating potential overstuffing. The lowest JST (0.81 mm) was calculated for the scapho-capitate joint. All other JST values were 2 mm or larger, as shown in Figure 5.

## 4. Discussion

Our analysis revealed no statistically significant volumetric differences between scaphoid models segmented from CT scans of frozen and thawed cadaveric wrists. This suggests that freezing and thawing cycles do not induce measurable morphological changes in the scaphoid bone, supporting the reliability of frozen cadaver specimens for accurate prosthesis modeling.

The initial “tight” segmentations, which represent the native bone surface, exhibited a slightly rough texture. This surface irregularity is likely due to two factors: the absence of the cartilage layer in CT imaging and inherent image noise affecting cortical bone segmentation. Since rough surfaces are undesirable in clinical use, smoothing techniques are applied during prosthesis design to ensure optimal articulation against adjacent bones.

The smoothing method adopted in our workflow introduced surface deviations of up to 0.64 mm when compared to the native segmentation. Importantly, these deviations remained below the minimum joint space thickness (JST) observed at the scapho-capitate interface (≥0.81 mm) and the cartilage layers of the distal radius, with values of 0.70 mm (±0.18) at the scaphoid fossa and 0.89 mm (±0.23) at the interfossal ridge [18]. These findings confirm that the smoothing process does not critically affect prosthesis fit within the anatomical joint space.

Interestingly, the scaphoid volumes obtained in our study—averaging 1257.42 mm^3^ (frozen) and 1259.22 mm^3^ (thawed)—are notably smaller than those reported by Pichler et al., who documented a mean volume of 3389.5 mm^3^ across 30 healthy scaphoids [11]. They also identified significant gender-based differences, with male scaphoids averaging 4057.87 mm^3^ compared to 2846.57 mm^3^ in females. This variation underscores the necessity for individualized prosthesis design, as highlighted in previous clinical studies [2].

**Figure 6:**
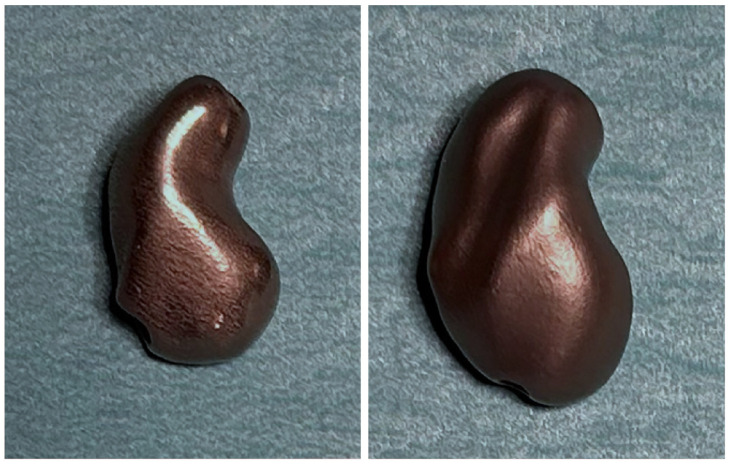
A 3D-printed scaphoid prosthesis (titanium, SLS) of a smaller woman (**left**) and a larger man (**right**).

Our own prosthesis design experiences echoed these interindividual differences, as we observed substantial size variations between male and female prosthetic models (Figure 6). These observations align with the findings of Heinzelmann et al., who emphasized the wide anatomical variability of the scaphoid [10].

While the sample size of this study is limited to a single cadaver specimen, we believe that the core question—whether freezing alters scaphoid size—has been adequately addressed within the scope of our experimental design. Extensive studies in the literature have already detailed interindividual anatomical variability, and our findings regarding freezing effects are consistent with prior studies reporting no dimensional changes in frozen–thawed long bones, skulls, lumbar spines, and cancellous bone specimens [3,4,5,6,7].

## 5. Conclusions

Our study demonstrated that freezing and thawing do not significantly affect the volume or shape of the cadaveric scaphoid, affirming the suitability of frozen specimens for patient-specific prosthesis modeling. Additionally, segmentation smoothing, which is essential for manufacturing-ready prosthesis surfaces, introduces only minor geometric deviations that remain within acceptable anatomical joint space ranges. These findings support the feasibility of using frozen cadaver wrists for accurate, reliable prosthesis design workflows. Further research using a larger sample size could support our findings.

## Figures and Tables

**Table 1:** Volumes of the frozen and thawed segmented native scaphoid polygon meshes.

	Frozen	Thawed	Difference
Scan ID	Volume [mm^3^]	Volume [mm^3^]	[%]
1	1254.32	1257.79	0.28
2	1253.31	1258.09	0.38
3	1261.33	1268.16	0.54
4	1265.97	1258.51	0.59
5	1273.02	1258.25	1.17
6	1249.78	1257.63	0.62
7	1259.22	1259.96	0.06
8	1246.46	1252.37	0.47
9	1253.58	1259.31	0.46
10	1257.34	1262.01	0.37
Average	1257.43	1259.22	0.14
SD	7.85	3.98	0.55

**Table 2:** Distance statistics (min/max/mean/sd) for absolute distances and the signed approach.

	Absolute Values for Heat Map	Signed Values for Heat Map
**Minimum [mm]**	0.00	−0.64
**Maximum [mm]**	0.64	0.53
**Mean [mm]**	0.17	0.01
**Standard Deviation**	0.11	0.2
**Included Numbers**	34,128	34,128

## Data Availability

The original contributions presented in this study are included in the article. Further inquiries can be directed to the corresponding author.
